# Picture quiz

**Published:** 2017-03-03

**Figure:**
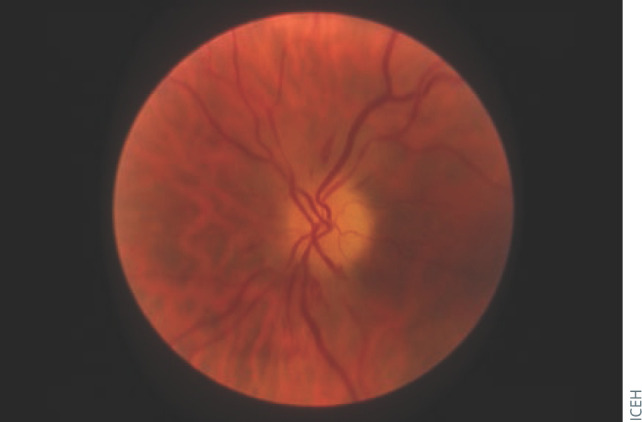


This 30-year-old woman presents with headache, nausea and confusion developing over the last 4 weeks. The appearance in both fundi is similar The visual acuity is 6/6 and 6/9. Pupils respond equally to light. Extraocular movements are full.

**Tick ALL that are TRUE**

**Question 1**
**The following signs are visible:**
  □ **a**. Hard exudates
  □ **b**. Intra-retinal haemorrhage
  □ **c**. Swelling of the disc
  □ **d**. Cotton wool spots
  □ **e**. Optic pallor/atrophy
**Question 2**
**The following are likely diagnoses:**
  □ **a**. Papillitis due to multiple sclerosis
  □ **b**. Hypertensive retinopathy
  □ **c**. Diabetic retinopathy
  □ **d**. Raised intracranial pressure
  □ **e**. Migraine
**Question 3**
**The following is indicated:**
  □ **a**. Lumbar puncture
  □ **b**. X-ray of the orbit
  □ **c**. Referral for brain imaging (MRI/CT scan)
  □ **d**. Careful history taking of medications
  □ **e**. Tensilon test.

## ANSWERS

1. Answer b and c. There is a swollen disc (disc edge not clearly visible) with a flame shaped haemorrhage above the disc superiorly.
2. Answer d. In papillitis the visual acuity is decreased. Hypertensive and diabetic retinopathy often have hard and soft exudates. Migraine would not last for so long. The symptoms of headache, nausea and confusion are typical of raised intracranial pressure (ICP) which is confirmed by the presence of swollen discs (papilloedema).
3. Answer c and d. Lumbar puncture should not be performed in the presence of papilloedma as it may cause “coning” and death. There is no evidence or orbital disease (no proptosis and normal ocular movements.) An MRI is indicated to look for an intra-cranial space occupying lesion. Some medications cause raised ICP so a careful history of medicine use is important. The Tensilon test is used to investigate myasthenia gravis which is not indicated here.

